# Does more support mean more literacy? The relationship and mechanisms between digital support and college students’ digital literacy

**DOI:** 10.3389/fpsyg.2025.1571926

**Published:** 2025-04-16

**Authors:** Ru Yan, Wenjing Hu

**Affiliations:** ^1^Institute of Education, Xiamen University, Xiamen, China; ^2^Baoding Preschool Teachers College, Baoding, China

**Keywords:** digital technology support, digital device support, digital literacy, self-efficacy, interpersonal interaction

## Abstract

**Introduction:**

The growing interest for digitization in education underscores the importance of students’ digital literacy. However, few previous studies have focused on the important role of digital support in college students’ digital literacy.

**Methods:**

Guided by the person-context interaction theory and based on a survey of 2,157 college students, this study aimed to reveal the effects of digital device support and digital technology support on college students’ digital literacy, and to further examine the mediating roles of self-efficacy and interpersonal interactions.

**Results:**

The results showed that both digital device support and digital technology support positively predicted digital literacy, and self-efficacy and interpersonal interaction mediated the relationship between digital device support and digital literacy. Self-efficacy mediated the relationship between digital technology support and digital literacy, while interpersonal interaction had a non-significant mediating effect in this relationship. Additionally, self-efficacy significantly predicted interpersonal interaction, forming a chain mediation effect between digital device support, digital technology support, and digital literacy.

**Discussion:**

This study explores the relationship between digital support and digital literacy in college contexts, emphasizing the important role of individual factors in this connection. The findings contribute to a systematic understanding of how environmental factors influence individual competence, provide empirical support for digital literacy research, and offer actionable insights for improving digital literacy in higher education. It is noteworthy that the research methods used in this study were based on self-reports, which may not accurately reveal causal relationships. Future research could improve the applicability and generalizability of the findings by adopting multimodal approaches.

## 1 Introduction

With the rise of the digital age, digital technology has become deeply integrated into all aspects of life. Its diversity and innovation have made it a central focus of academic research (Farias-Gaytan et al., 2023; Muslim and Setyono, 2024; Quraishi et al., 2024). As digitalization continues to shape various sectors, education systems are increasingly prioritizing the development of students’ digital literacy. College students, as key members of future societies, are not only primary beneficiaries but also active participants in the digitalization process. Their ability to harness strong digital skills is essential for both their personal development and societal advancement. Digital literacy is a comprehensive ability that enables individuals to effectively acquire, evaluate, apply, and create information within a digital environment (Reddy et al., 2020). It encompasses four core dimensions: technical skills, critical thinking, ethical practice, and social responsibility. Specifically, it includes the ability to utilize technical skills and conduct information retrieval, as well as assess online content, understand algorithms, address ethical considerations related to privacy and fairness, and cultivate digital citizenship awareness, thereby highlighting the individual’s influence within the digital ecosystem (Park et al., 2021; Spires et al., 2019; Tinmaz et al., 2022; Wuyckens et al., 2022). College students with high levels of digital literacy are better equipped to meet the demands of the digital age, improve their learning in digital environments, and enhance their core competencies, thus laying a solid foundation for their future careers (Porat et al., 2018). For this reason, it is of great importance to focus on the digital literacy of college students.

Previous research on digital literacy has primarily focused on specific contexts, such as blended learning environments and formal online education (Chiu, 2021; Yustika and Iswati, 2020). Some studies have examined digital literacy as an antecedent variable influencing individual and societal development (Bejaković and Mrnjavac, 2020; Vissenberg et al., 2022). However, fewer studies have investigated the critical role of digital support on campuses in improving students’ digital literacy. The person-context interaction theory suggests that individual behavior and development cannot be understood in isolation from the context; rather, individuals and their contexts shape outcomes through continuous interaction (Magnusson and Stattin, 1998). The theory emphasizes that individual behavior and development must be understood in relation to the environment, just as the development of digital literacy inevitably unfolds in the interconnected and pervasive digital environment (Chan, 2022). Thus, this theory can comprehensively explain the development of digital literacy within the context of digital support, providing us with a robust theoretical perspective. Digital literacy is not only the result of individual effort but also benefits from a robust environment of technological support and rich educational resources (Diseiye et al., 2024), which provides more learning opportunities and enhances individuals’ understanding and application of new technologies, thereby fostering the development of digital literacy (Audrin and Audrin, 2022). In today’s digitally diverse and resource-rich society, digital support in colleges, whether in the form of equipment or technological assistance, creates a favorable environment that significantly enhances students’ digital skills (Morgan et al., 2022). Furthermore, the theory also posits that the environment, as a distal factor, influences individual development through proximal factors (Magnusson and Stattin, 1998). Among these factors, self-efficacy represents an internal individual characteristic, reflecting one’s confidence and belief in their digital abilities within a digital environment (Ulfert-Blank and Schmidt, 2022). Conversely, interpersonal interaction constitutes an external individual factor, reflecting how an individual communicates and interacts with others in their academic and daily life (Mehall, 2020). These two factors are critical for college students. Therefore, self-efficacy and interpersonal interaction could serve as key mediators between environmental influences and individual development. However, a paucity of research has characterized the exploration of the relationship and underlying mechanisms between college digital support and students’ digital literacy. This study aims to fill this gap by exploring both the relationship and underlying mechanisms involved. Given the exploratory nature of the early stages of the research, this study emphasizes examining the individual subjective cognitive level through self-assessment methods. Although this approach cannot delineate the causal relationships involved, it can still reveal correlations to some extent, providing a starting point and foundation for future longitudinal research and experimental exploration.

## 2 Literature review

### 2.1 Digital support and college students’ digital literacy

Digital support is defined as the comprehensive institutional services related to digital education provided to college students. This includes hardware support, which refers to the availability of digital learning equipment supplied by colleges, such as computers, digital learning tools, software, and digital audio equipment. Additionally, it encompasses digital technical support aimed at training students in the use of various digital tools, including network application technology, software operation, and different forms of digital literacy training (Park, 2011). Furthermore, the concept can be expanded to include digital policy support from colleges as well as assistance from faculty and peers (Chiu, 2021; Strobl et al., 2019). Overall, digital support can significantly enhance students’ learning experiences and overall digital literacy. To the best of our knowledge, no previous research has directly revealed the relationship between digital support in higher education and digital literacy of college students. Relevant studies suggest that digital support systems promote student engagement in multimedia information processing by enriching learning environments, enhancing emotional involvement, and fostering enjoyable learning experiences (Chiu, 2021). Other studies have demonstrated the importance of digital devices, such as mobile-enabled creation platforms (e.g., iPad, iMovie) play a pivotal role in developing multimodal digital narratives, with demonstrated efficacy in advancing students’ technological competencies and critical digital literacies (Churchill, 2020). Similarly, digital device functionality is increasingly recognized as a critical dimension of digital media literacy, as the complexity of digital literacy requires the support of a variety of tools (Park, 2011). Another teacher-based study found that manipulating information and communications technology (ICT) plays a crucial role in the development of teachers’ digital literacy, with mastery of app manipulation techniques particularly improving teachers’ digital literacy (Sulasmi, 2022). Therefore, both digital technology support and digital device support are closely related to the digital literacy of college students.

However, the provision of physical equipment and tools constitutes the primary focus of digital device support, while the provision of training in the skills required to use these resources constitutes the primary focus of digital technology support. These two forms of support may be related to the development of digital literacy in college students to varying degrees. Examining digital device support and digital technology support within a unified framework will facilitate a more comprehensive understanding of their combined and individual impact on students’ digital literacy. This analysis can also inform the development of more effective support and services to enhance students’ competence in digital environments. Therefore, this study will first examine the relationship between institutional support and digital technology support and students’ digital literacy, and further compare whether there are any differences.

### 2.2 The mediating role of self-efficacy and interpersonal interaction

Person-context interaction theory (PCIT) states that the environment, as a distal factor, indirectly influences an individual’s development by affecting their proximal factors (Magnusson and Stattin, 1998). Within this theoretical framework, self-efficacy and interpersonal interactions, as two key personal factors, may mediate the relationship between digital supports in colleges and students’ digital literacy. Self-efficacy refers to an individual’s belief and confidence in their ability to successfully accomplish a particular task or cope with a particular situation (Bandura, 1994; Trusz and Bąbel, 2016; Schwarzer and Luszczynska, 2008). Interpersonal interaction refers to the process of communication and mutual influence among college students, both on and off campus, through various forms and channels (Kumari and Verma, 2015; Turner, 1988).

#### 2.2.1 The role of self-efficacy

Self-efficacy may play a mediating role in the relationship between digital support and digital literacy among college students. Digital supports (e.g., online learning platforms, digital resources, and technology training) may be associated with students’ levels of self-efficacy. Digital device support and digital technology support work synergistically and are closely related to college students’ self-efficacy. Digital device support, especially when students use school-provided digital resources, including online libraries, learning management systems, and virtual labs, prompts students to show higher academic achievement and self-efficacy (Kay and Lauricella, 2011). The efficacy of digital devices and tools in facilitating the management of learning tasks has been demonstrated to enhance students’ confidence and perceived competence, thereby leading to substantial improvements in their learning experience and self-efficacy (Anthony, 2008; Johnson and Howell, 2005). At the same time, digital technology support provided by colleges may also promote self-efficacy among students. A series of related studies have identified that appropriate technical support and training promotes teachers’ mastery of digital technologies, thereby contributing to their self-efficacy (Scherer et al., 2019; Teo et al., 2015). A similar finding emerged from a study on language learning found that mastery of educational technology could increase the self-efficacy of students learning English as a foreign language by improving their interactive and dynamic thinking (Zhang, 2022).

In addition, self-efficacy further promotes digital literacy among college students. Previous research has indicated that students who possess high self-efficacy in digital learning environments exhibit increased confidence and skill in the utilization of digital tools and technologies, along with higher levels of knowledge in the selection of digital resources (Tang and Tseng, 2013). Self-efficacy has also been shown to increase students’ motivation, self-directed learning ability, and persistence in learning, thereby effectively enhancing mobile learning (Bettayeb et al., 2020). In a study on professionals, self-efficacy was found to exert a positive and significant effect on digital literacy. Higher self-efficacy led to greater self-confidence in mastering skills, which improved individuals’ adaptability in digital environments (Nusannas et al., 2020).

#### 2.2.2 The role of interpersonal interaction

Interpersonal interactions may play a mediating role in the relationship between digital support and digital literacy among college students. Digital technology plays an increasingly prominent role in shaping modern social interactions (Satata et al., 2023). Previous studies have demonstrated that the introduction of digital devices significantly enhances the frequency of interactions in technology classrooms by enabling students to engage in discussions anytime and anywhere, while also increasing the diversity of interaction partners and enriching the overall content of these interactions (Wang et al., 2016). In a similar vein, institutions of higher education have the capacity to leverage digital devices and online platforms to provide students with expanded opportunities for communication and interpersonal interaction. These resources have been shown to promote communication and social activities within the campus environment, enhance connections among students, and foster a sense of cohesion (Inkpen et al., 2002). The role of digital technology support in influencing college students’ interpersonal interactions has been inconsistent in several studies. Some scholars believe that increased digital technology support will promote interaction among students, especially mastery of digital technology facilitates the creation of collaborative learning environments and simplifies cooperation and communication, thus promoting interaction and teamwork (Han and Son, 2020). Conversely, other researchers have argued that excessive use of technology may reduce interpersonal interactions, as students with digital technology mastery may invest more time on digital platforms, potentially hindering face-to-face communication (Ivan, 2023; Ruben et al., 2021).

Furthermore, interpersonal interactions may contribute to the digital literacy of college students. Previous research has found that students in a college setting can master new digital tools and technologies more quickly and effectively by communicating and interacting with their peers and sharing experiences and skills in the use of digital technologies (Lambert and Corrin, 2007). Interpersonal interaction provides a platform for students to share resources and tools, thereby enabling them to learn about and access a variety of useful learning materials, software tools, and technical know-how. Effective interpersonal interaction can help students solve technical problems, share resources and tools, and improve their technical skills (Ng, 2012).

#### 2.2.3 Relationship between self-efficacy and interpersonal interaction

In addition, self-efficacy and interpersonal interactions may form a chain-mediated mechanism in the relationship between digital support and digital literacy among college students. In other words, self-efficacy may further influence interpersonal interactions. Relevant literature states that individuals deficient in self-efficacy frequently exhibit diminished confidence in their social aptitude, apprehension regarding unfavorable assessments, and an inclination toward heightened social pressure, which results in social evasion (Matsushima and Shiomi, 2003). Compared to those who lack self-efficacy, individuals with high self-efficacy show greater courage in social interactions, allowing them to manage various scenarios and demonstrate higher social competence compared to those with low self-efficacy (Locke and Sadler, 2007). Meanwhile, people with high self-efficacy are more proactive in social interactions, establish and maintain positive relationships, and receive positive affective feedback from social interactions (Hullman et al., 2010). Based on the above, this study will further explore the chain-mediated mechanism of self-efficacy and interpersonal interaction.

### 2.3 The current study

**Figure 1:**
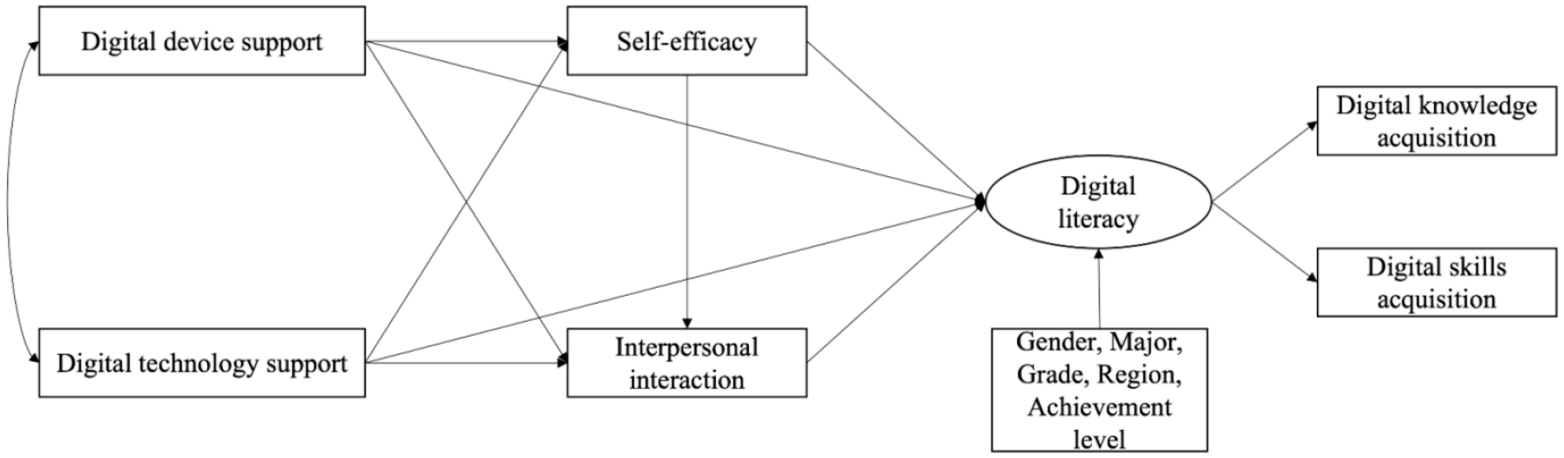
Hypothesized model.

This study explores the relationship between digital support on college students’ digital literacy based on the theory of person-context interaction theory, and further examines the mediating roles of self-efficacy and interpersonal interactions. This study controls for gender, major, grade, academic performance, and region of college students in the data analysis. The specific hypothesized model of this study is shown in Figure 1. The hypothesized path for this study is shown in Table 1.

**Table 1:** All hypothetical paths of this study.

Hypotheses	Path
H1a	Digital device support → Digital literacy.
H1b	Digital technology support → Digital literacy.
H2a	Digital device support → Self-efficacy → Digital literacy.
H2b	Digital technology support → Self-efficacy → Digital literacy.
H3a	Digital device support → Interpersonal interaction → Digital literacy.
H3b	Digital technology support → Interpersonal interaction → Digital literacy.
H4a	Digital device support → Self-efficacy → Interpersonal interaction → Digital literacy.
H4b	Digital technology support → Self-efficacy → Interpersonal interaction → Digital literacy.

## 3 Methods

### 3.1 Study participants and research procedures

This study conducted an online survey by recruiting college students for voluntary participation, using a set of digital literacy questionnaires for college students. Over a period of 3 months, a total of 2,157 valid samples were collected from nine provinces in China. This study recruited participants from both vocational colleges and regular undergraduate institutions. The sample included current college students from freshman to senior year, with 1,567 freshmen, 432 sophomores, 126 juniors, and 32 seniors. The proportion of juniors and seniors in the sample is relatively low, primarily due to conflicts between their academic and career planning timelines. However, there is still a small portion of upperclassmen participating in this study, which maintains a certain level of exploratory potential and applicability. Among them, male students accounted for 43.2%, while female students accounted for 56.8%; urban students accounted for 31.6%, while rural students accounted for 68.4%; humanities accounted for 4.2%, social sciences accounted for 35.4%, science accounted for 14.5%, and industry, agriculture, military and medicine accounted for 45.9%, ensuring a balanced distribution of the sample.

The procedure of this survey was designed to assess the digital literacy of college students and the factors influencing it, and was first approved by the college Ethics Committee to ensure compliance with ethical standards. Through collaboration with the cooperating teacher, the purpose of the study was presented in class, and students were invited to participate. Before completing the questionnaire, all participants were informed in detail about the purpose of the study, the process, the potential risks, and data usage. They were required to sign an informed consent form confirming their voluntary participation and acknowledgement of their rights. During the survey period, the questionnaire was released through an online platform for efficient data collection.

### 3.2 Measurement tools

#### 3.2.1 Digital support scale

By interviewing students about their perceptions of the digital environment, this study identified the main components of digital environment perception. A self-administered scale was designed, which consisted of six question items covering two dimensions. Among them, digital device support in higher education contains three question items, such as “the school can provide sufficient hardware equipment to meet digital learning needs.” Digital technology support in colleges consisted of three items, such as “The school has specialized personnel (full-time staff) to help with digital learning.” Validated factor analysis was used to examine the model fit of the scale structure. The results showed that all of them were saturated models, and the scale had good structural validity. The results of CFA were as follows: *χ^2^* / *df* = 6.033 (*p* = 0.000), RMSEA = 0.048, CFI = 0.997, TLI = 0.994, SRMR = 0.007. The Cronbach’s alpha coefficients in this study were 0. 906 for digital device support and 0. 887 for digital technology support.

#### 3.2.2 Digital literacy scale

Digital literacy was measured using a scale developed by Ng (2012) consisting of nine items, such as “I know how to solve technical problems I encounter” and “I can easily learn new technical tools.” Responses were given on a five-point Likert scale ranging from 1 “strongly disagree” to 5 “strongly agree.” Higher scores indicate higher digital literacy. The results of CFA analysis showed that the scale had good construct validity with *χ^2^* / *df* = 7.210 (*p* = 0.000), RMSEA = 0.054, CFI = 0.996, TLI = 0.990, SRMR = 0.009. The Cronbach’s alpha coefficient for this scale in this study was 0. 970.

#### 3.2.3 Self-efficacy scale

Self-efficacy was measured using a scale developed by Shen et al. (2013) and others. The scale consists of eight items, for example, “I can complete a variety of study programs (e-learning programs, blended learning programs, etc.) with good grades,” and “I am able to understand complex concepts.” Responses were on a five-point Likert scale ranging from 1 “strongly disagree” to 5 “strongly agree.” The results of CFA analysis showed good construct validity with *χ*^2^ / *df* = 5.478 (*p* = 0.000), RMSEA = 0.046, CFI = 0.998, TLI = 0.994, SRMR = 0.005. The Cronbach’s alpha coefficient for this scale in this study was 0. 973.

#### 3.2.4 Interpersonal interaction scale

The Interpersonal Interaction Scale was adopted from the scale developed by Shen et al. (2013) and others. The scale had 5 items and was scored on a 5-point Likert scale ranging from 1 “strongly disagree” to 5 “strongly agree.” Specific questions included, for example, “Initiating social interactions with classmates” and “Interacting with other students in a respectful manner.” The results of CFA analysis showed that the scale had good construct validity: *χ*^2^ / *df* = 5.622 (*p* = 0.000), RMSEA = 0.046, CFI = 0.999, TLI = 0.995, SRMR = 0.004. The Cronbach’s alpha coefficient for this scale in this study was 0. 927.

The standardized estimate range, CR, and AVE are shown in Table 2.

**Table 2:** Standardized estimate range, CR, and AVE for each variable.

Variable name	Item count	Standardized estimate range	CR	AVE
1. Digital support	6	0.749 ~ 0.908	0.941	0.728
2. Digital literacy	9	0.836 ~ 0.922	0.969	0.778
3. Self-efficacy	8	0.869 ~ 0.936	0.972	0.810
4. Interpersonal Interaction	5	0.773 ~ 0.881	0.923	0.707

In this study, CR > 0.7 and AVE > 0.5 are considered ideal thresholds.

### 3.3 Data analysis

SPSS 25.0 and Mplus 8.3 software were used to analyze the data in this study. Data were organized by calculating descriptive statistics and Pearson’s correlation to test the association between the main variables. Structural equation modeling (SEM) was constructed. The chi-square test, comparison of CFI, TLI, RMSEA and SRMR were used to test the model fit. The bootstrap method was adopted to test the mediation effect.

## 4 Results

### 4.1 Correlation analysis of main variables

The means, standard deviations and correlations of all variables are shown in Table 3. All core variables had a significant positive two-by-two relationship (*p* < 0.001). Based on the results of the correlation analysis, the hypothesized relationships between the variables can be further tested subsequently using structural equation modeling.

**Table 3:** Correlation table of core variables.

	1	2	3	4	5	6
Digital technology support	–					
Digital device support	0.879^***^	–				
Digital knowledge acquisition	0.623^***^	0.600^***^	–			
Digital skills acquisition	0.619^***^	0.600^***^	0.949^***^	–		
Self-efficacy	0.621^***^	0.597^***^	0.819^**^	0.833^***^	–	
Interpersonal interaction	0.563^***^	0.554^***^	0.669^***^	0.697^***^	0.751^***^	–
*M*	4.024	4.060	3.709	3.785	3.909	4.154
*SD*	0.954	0.952	0.994	0.973	0.920	0.832

***p < 0.001.

### 4.2 Structural equation modeling results

**Figure 2:**
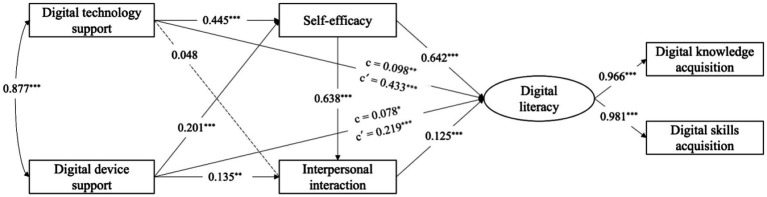
Structural equation result model.

Firstly, the predictive effect of college digital support on students’ digital literacy was tested through structural equation modeling. The results of this study can be further referenced in Figure 2, specifically regarding the two direct pathways (c’). The model fit indices were: *χ^2^* / *df* = 3.262 (*p* = 0.000), RMSEA = 0.032, CFI = 0.998, TLI = 0.995, SRMR = 0.004, indicating good fit. Both digital technology support (standardized *β* = 0.433, *p* < 0.001) and digital device support (standardized *β* = 0.219, *p* < 0.001) positively predicted college students’ digital literacy.

Secondly, digital self-efficacy and interpersonal interaction were included as mediators in the model for testing. The results of the mediating paths can be further referenced in Figure 2. The model fit indicators were: *χ^2^*/*df* = 5.811 (*p* = 0.000), RMSEA = 0.046, CFI = 0.988, TLI = 0.985, SRMR = 0.053, demonstrating good fit. The path analysis revealed significant relationships among the variables, with effect sizes quantified using Cohen’s *f*^2^ (Table 4). Digital technology support had a significant positive effect on digital literacy (*β* = 0.098, *f*^2^ = 0.004), while digital device support also showed a significant positive association with digital literacy (*β* = 0.078, *f*^2^ = 0.004). Digital technology support significantly predicted self-efficacy (*β* = 0.445, *f*^2^ = 0.076) but had no significant effect on interpersonal interaction (*β* = 0.048). Digital device support significantly influenced both self-efficacy (*β* = 0.201, *f*^2^ = 0.015) and interpersonal interaction (*β* = 0.135, *f*^2^ = 0.002). Self-efficacy significantly predicted interpersonal interaction (*β* = 0.638, *f*^2^ = 0.581) and digital literacy (*β* = 0.642, *f*^2^ = 0.618), while interpersonal interaction also contributed significantly to digital literacy (*β* = 0.125, *f*^2^ = 0.023).

**Table 4:** Summary table of Cohen’s *f*^2^ for all paths.

Path	*β*	*p*	Full *R*^2^	Reduced *R*^2^	Cohen’s *f*^2^
Digital technology support → Self-efficacy	0.445	<0.001	0.396	0.350	0.076
Digital device support → Self-efficacy	0.201	<0.001	0.396	0.387	0.015
Self-efficacy → Interpersonal interaction	0.638	<0.001	0.578	0.333	0.581
Digital technology support → Interpersonal interaction	0.048	>0.050	0.578	0.578	0.000
Digital device support → Interpersonal interaction	0.135	<0.010	0.578	0.574	0.002
Self-efficacy → digital literacy	0.642	<0.001	0.741	0.581	0.618
Interpersonal interaction → digital literacy	0.125	<0.001	0.741	0.735	0.023
Digital device support → digital literacy	0.078	<0.050	0.741	0.740	0.004
Digital technology support → digital literacy	0.098	<0.010	0.741	0.740	0.004

β = Standardized β.

The effect sizes varied substantially across paths. Self-efficacy demonstrated the largest effects, with *f*^2^ values of 0.618 (on digital literacy) and 0.581 (on interpersonal interaction), both classified as large effects. Digital technology support’s impact on self-efficacy (*f*^2^ = 0.076) and digital device support’s influence on self-efficacy (*f*^2^ = 0.015) represented small-to-moderate effects, respectively. Most direct predictors of digital literacy (e.g., interpersonal interaction, digital device/technology support) exhibited very small effects (*f*^2^ ≤ 0.023). These findings highlight self-efficacy as the central driver in the model, with both direct and indirect effects overshadowing other pathways.

The bias-corrected percent bootstrap method (5,000 replicate samples) was used to test the mediation effect for the six pathways, as shown in Table 5. The 95% confidence interval for Digital technology support → Interpersonal interaction → Digital literacy includes 0, indicating that the mediating effect is not significant, and this result does not support H3b. In contrast, the 95% confidence intervals for the other relationships do not include 0, suggesting that the mediating effects are significant, thus supporting H2a, H2b, H3a, H4a, and H4b.

**Table 5:** Testing the mediating effect.

Model pathways	Effects sizes	95% CI	
		Lower Upper	
Digital device support → Self-efficacy → Digital literacy	0.129^***^	0.062	0.191
Digital device support → Interpersonal interaction → Digital literacy	0.017^*^	0.006	0.033
Digital technology support → Self-efficacy → Digital literacy	0.286^***^	0.225	0.348
Digital technology support → Interpersonal interaction → Digital literacy	0.006	−0.004	0.019
Digital device support → Self-efficacy → Interpersonal interaction → Digital literacy	0.016^**^	0.009	0.029
Digital technology support → Self-efficacy → Interpersonal interaction → Digital literacy	0.036^***^	0.022	0.054

## 5 Discussion

This study explores the relationship between digital support in higher education and college students’ digital literacy, and examines the mediating roles of self-efficacy and interpersonal interaction. It not only supports the person-context interaction theory, but also provides empirical support for further understanding the relationship and mechanisms between digital device support, digital technology support in higher education, and digital literacy.

### 5.1 Digital support and college students’ digital literacy

The results of this study found that digital support in higher education, including both digital technology support and digital device support, all are related to the digital literacy of college students. The findings fill the gap in previous research on this topic. Colleges provide students with digital device support, including online learning platforms, rich online courses, and learning resources, enabling students to access knowledge at any time and in any location. They also organize webinars, online tutorials, and virtual labs to enhance students’ hands-on abilities (Gikas and Grant, 2013). Moreover, the provision of students with the necessary equipment, including high-performance computers, tablets, and smartphones, in addition to the equipping of said students with virtual reality and augmented reality devices to enhance the interactivity and immersion of learning (Zhang et al., 2014), can significantly enhance students’ digital literacy. Furthermore, the provision of digital technology support in academic institutions primarily encompasses the delivery of pertinent training programs, which aim to equip students with the necessary skills to utilize various digital instruments and software, including programming languages and data analysis tools (Henderson et al., 2017). Through studying such courses and participating in various types of training, students are able to acquire digital operation ability and improve digital skills. Concurrently, numerous institutions of higher education have established technical support centers with the objective of providing students with timely assistance and advice. These centers aim to facilitate the students’ navigation of technical challenges and enhance their proficiency in the utilization of digital tools. These measures enable students to confidently apply digital learning tools in academic research and daily study, thereby improving their digital literacy (Sulasmi, 2022).

### 5.2 Mediating role of self-efficacy and interpersonal interactions

The results of this study found that self-efficacy mediates the relationship between digital support and digital literacy. In higher education, providing digital technology support to students is related to their higher levels of self-efficacy. Systematic training helps college students become proficient in using various digital tools, making them more confident when facing digital tasks. When college students encounter technical problems, timely and effective technical support reduces their frustration, encourages them to feel more secure in using digital resources, and increases their trust in their own abilities (Zhang, 2022). At the same time, the provision of digital device support by higher education institutions is also related to the development of students’ self-efficacy. High-performance digital devices reduce technological barriers and allows students to focus more on their studies, thus increasing self-efficacy (Kay and Lauricella, 2011). Using advanced devices for learning and experimentation allows students to feel empowered. Therefore, both digital technology support and digital device support may be significant correlates of college students’ self-efficacy levels. Furthermore, the development of self-efficacy is closely related to the development of college students’ digital literacy. Students with higher self-efficacy typically exhibit greater motivation and commitment to learning (Alemayehu and Chen, 2023), prompting them to actively explore and utilize digital technologies and resources, thereby enhancing their digital literacy. Self-efficacy can also help students maintain a positive attitude towards learning (Pan, 2020), and develop an interest and enthusiasm for digital skills as they continue to experiment and practice.

This study also found that interpersonal interactions mediate the relationship between digital device support and students’ digital literacy. Digital device support is related to interpersonal interactions. This phenomenon may be attributed to the proliferation of advanced digital devices, which facilitate seamless collaboration and communication among students (Zarzycka et al., 2021). Advanced devices support multimedia communication tools such as videoconferencing software, social media platforms, and online discussion boards, enhancing interactions among students (Inkpen et al., 2002). Better interpersonal interactions are associated with higher digital literacy among college students. Efficient interpersonal interactions facilitate skill transfer and mutual learning. For example, skilled students can support others in using digital devices, thereby enhancing overall digital literacy. Peer guidance and mutual support through interactions also promote individual digital literacy.

However, the mediating role of interpersonal interaction in the relationship between digital technology support and college students’ digital literacy was not significant. There may be several reasons for this phenomenon. First, digital technology support emphasizes independent and personalized learning, prompting students to operate and explore independently. This mode of independent learning reduces students’ need for help from others in the learning process, making interpersonal interaction less necessary and thus not significant in the process of enhancing digital literacy. Second, modern digital technologies are designed to be intuitive and user-friendly, enabling students to use them independently with ease. As a result, reliance on others’ help and communication decreases, diminishing the role of interpersonal interaction in mastering digital technologies. Third, technical support services are usually based on individualized help, which reduces the opportunities for interaction between students, rendering the mediating role of interpersonal interaction in enhancing digital literacy insignificant.

### 5.3 Chain mediation of digital self-efficacy and interpersonal interaction

The results of this study also found that self-efficacy and interpersonal interactions chain-mediated the relationship between digital support and college students’ digital literacy. That is, higher self-efficacy is associated with better interpersonal interactions among college students. Students with high self-efficacy have higher confidence in their abilities and task completion, which makes them more willing to engage in interpersonal interactions (Matsushima and Shiomi, 2003). Such students are more willing to initiate and participate in interactive activities, such as group discussions and collaborative projects, because they believe that their abilities add value to the team (Hullman et al., 2010). Additionally, students with strong self-efficacy are more inclined to share their knowledge and experiences, which not only fosters their own learning but also facilitates group interactions. This sense of self-efficacy enhances students’ confidence and motivation to address challenges, further promoting positive and constructive behaviors in their interpersonal interactions. Therefore, digital device support is linked to increased self-efficacy. In turn, higher self-efficacy fosters interpersonal interactions, ultimately enhancing college students’ digital literacy.

Given the survey participants in this study, while some upperclassmen participated, the participants were primarily freshmen. The results of this study are of greater significance to college freshmen. Because freshmen are in a critical period of adapting to college life, their experiences with digital device support and digital technology support are closely related to their level of digital literacy. Some of these early experiences are important processes in their adaptation to an increasingly digital environment. Self-efficacy and interpersonal interactions also play a potentially mediating role in this process. Self-efficacy enhances freshmen’s motivation to use digital technology and promotes skill enhancement. And interpersonal interactions, such as cooperation and communication among classmates, can further strengthen digital literacy.

### 5.4 Research implications and limitations

The findings of this study carry significant educational implications for both educators and students. Firstly, universities should adopt a comprehensive approach to digital skills training by providing foundational courses that cover essential programming and data analysis skills, along with specialized workshops focused on advanced topics such as machine learning and big data. Regularly updating the curriculum to reflect the latest technological trends is also vital. Additionally, establishing a technology support center to offer immediate assistance is crucial. Secondly, universities should ensure that students have access to high-performance digital devices by offering low-cost rental or financing options for laptops and tablets. Introducing virtual reality (VR) and augmented reality (AR) technologies, along with relevant training, can further enhance the learning experience. Optimizing device usage strategies based on student feedback will help maximize the effectiveness of these tools. Thirdly, fostering collaboration and interactive learning is essential. Universities can facilitate group projects through online platforms, organize interdisciplinary experiments and innovation competitions to expand students’ knowledge and practical skills, and implement incentives for knowledge sharing and teamwork to encourage effective peer support. Finally, enhancing students’ self-efficacy and providing psychological support are critical. This can be achieved by offering constructive feedback to boost confidence, providing counseling services to help maintain motivation, and creating supportive communities and alumni mentoring programs to promote interpersonal relationships and career development.

In addition, there are some limitations to this study. Firstly, the survey relies on self-report data from university students, lacking objective assessments of digital literacy. Future research should incorporate information from multiple sources and include behavioral measurement indicators, such as practical tests, programming exercises, or digital problem-solving tasks, to validate the self-report findings. This approach would provide a more comprehensive understanding and enhance the rigor and reliability of the research results. At the same time, due to the limitations of random sampling, the participants in this study were predominantly college freshmen, with only a small number of upperclassmen involved, which enhances the generalizability of the findings among freshmen. Future research should employ cluster stratified sampling to balance the samples across different groups, thereby ensuring the universal applicability of the research results. Secondly, the cross-sectional design of this study does not truly reveal complex causal relationships. We consider using a longitudinal tracking design in future research to follow the survey population over a certain period of time in order to more clearly explore the causal relationships and mechanisms. Additionally, due to the limitations of the survey design, potential confounding variables such as socioeconomic status, prior digital experience, and attitudes toward technology culture were not excluded, which may have impacted the robustness of the results to some extent. In future research, this can be addressed by enhancing the questionnaire design and expanding the sample collection to control for confounding variables. Finally, this study was conducted in only nine provinces of China and does not yet possess national representativeness. Furthermore, given the context dependency of digital literacy, the findings may not be generalized to educational systems in Western or other developing countries. Future studies will seek global collaborators to expand the scope of the investigation, thereby enhancing the universality and generalizability of the results.

## Data Availability

The raw data supporting the conclusions of this article will be made available by the authors, without undue reservation.
